# A novel role for the obesity-associated gene FTO in ciliogenesis and Wnt signalling

**DOI:** 10.1186/2046-2530-1-S1-P75

**Published:** 2012-11-16

**Authors:** DPS Osborn, S Mukherjee, RM Roccasecca, I Barosso, D Stemple, PL Beales, S Christou-Savina

**Affiliations:** 1 UCL Institute of Child Health, UK; 2 Wellcome Trust Sanger Institute, UK

## 

Common intronic variants in hFTO, an oxoglutarate-dependent dioxygenase, were identified in GWAS to cause a predisposition to obesity. Inactivation of Fto in human and mouse models results in reduced viability, postnatal growth retardation, enhanced metabolism and loss of adipose tissue. However, the function of Fto during development and obesity remains unclear. We sought to clarify the function of Fto using zebrafish and cell culture. In zebrafish, we found loss of *fto* results in a ciliopathy-like phenotype. Morphants display small eyes, curved tails, otolith defects, disorganised melanocytes, *situs inversus* and renal cysts. Concurrently, cilia were absent in the olfactory bulbs and disorganised in the pronephric ducts (PND) and neural tube. Functional analysis of motile cilia in the PND and Kupffer’s vesicle confirmed a direct ciliary defect. Wnt signalling has been implicated in adipogenesis and therefore we decided to investigate whether Fto may interact at this level. We found that β-Catenin and downstream target genes are consistently downregulated in *fto* morphants, HEK293T cells transfected with an shRNA Fto expressing plasmid, and *Fto-/-* MEFs. Furthermore, β-Catenin failed to translocate to the nucleus in *Fto-/-* MEFs in response to Wnt3a stimulation. By completing a comparative Wnt signalling phospho-antibody array, on *Fto*+*/*+ and *Fto-/-* MEFs, we identified increased PKC and CamKII activity: two non-canonical/Ca2+ Wnt components. Indeed, phospo-CamkII is up regulated in *fto* morphants and in response to Wnt3a in *Fto-/-* MEFs. Taken together, these data show that Fto acts as a key cilia-mediated regulator of canonical and non-canonical/Ca2+ Wnt signalling.

